# Retraction Note to: Approaching treatment for immunological rejection of living-donor liver transplantation in rats

**DOI:** 10.1186/s12876-022-02450-3

**Published:** 2022-08-15

**Authors:** Yanhu Feng, Zhijian Han, Zedong Feng, Bofang Wang, Huijuan Cheng, Luxi Yang, Yangbing Li, Baohong Gu, Xuemei Li, Yahao Li, Yumin Li, Chen Wang, Hao Chen

**Affiliations:** 1 Department of Gastroenterology, Lanzhou University Second Hospital, Lanzhou, 730030 China; 2 Key Laboratory of the Digestive System Tumors of Gansu Province, Lanzhou University Second Hospital, Lanzhou, 730030 China; 3 Medical School of Lanzhou University, Lanzhou, 730030 China; 4 Department of Tumor Surgery, Lanzhou University Second Hospital, Lanzhou, 730030 China; 5 Department of General Surgery, Lanzhou University Second Hospital, Lanzhou, 730030 China

## Retraction Note: BMC Gastroenterology (2020) 20:7 https://doi.org/10.1186/s12876-019-1130-x

The Editor has retracted this article because Figs. 2 and 3 contain image panels that appear in figures published in another article [1], as detailed below:

- Figure 2 (SI panel) is present in [1] as Fig. 3a (panel SFS at 30 min);
- Figure 3 (panel TUNEL WAT) is present in [1] as Fig. 4b [panel SFS PCNA (1 day)];
- Figure 3 (panel PCNA WAT) is present in [1] as Fig. 4b [panel TPIPSS TUNEL (1 day)];
- Figure 3 (panel PCNA SAT) is present in [1] as Fig. 4b [panel SFS TUNEL (1 day)];
- Figure 3 (panel PCNA SATa) is present in [1] as Fig. 4b [panel TPIPSS PCNA (1 day)].

The Editor therefore considers the data reported in this article to be unreliable.

Hao Chen, Bofang Wang and Yanhu Feng agree to this retraction. The other authors have not responded to any correspondence from the editor/publisher about this retraction.
